# 血友病治疗中国指南（2025年版）

**DOI:** 10.3760/cma.j.cn121090-20250729-00354

**Published:** 2025-08

**Authors:** 中华医学会血液学分会血栓与止血学组 中国血友病协作组

## Abstract

血友病是一种Ⅹ染色体连锁的隐性遗传性出血性疾病，主要由凝血因子Ⅷ（FⅧ）或凝血因子Ⅸ（FⅨ）活性降低或缺乏所致。根据缺乏的凝血因子不同，可分为血友病A（FⅧ缺乏）和血友病B（FⅨ缺乏）两类，分别由相应凝血因子基因突变引起，其中血友病A最常见，约占血友病总数的80%。近年来，随着长效凝血因子产品、非因子类产品及基因治疗等治疗手段的问世，血友病已进入新型治疗时代，部分产品已在我国上市，显著改善了患者的治疗效果。为规范新型治疗方式及治疗理念下的诊疗行为，中华医学会血液学分会血栓与止血学组与中国血友病协作组共同制定了本指南。

## Abstract (en)

Hemophilia is an X-linked recessive inherited hemorrhagic disease primarily caused by reduced activity or deficiency of coagulation factor Ⅷ（FⅧ）or coagulation factor Ⅸ（FⅨ）. Depending on the specific coagulation factor deficiency, it is classified into two types: hemophilia A（caused by FⅧ deficiency）and hemophilia B（caused by FⅨ deficiency）, each attributed to gene mutations in their respective coagulation factors. Among these, hemophilia A is the most prevalent, accounting for approximately 80％ of all hemophilia cases. In recent years, the advent of novel therapeutic strategies—including long-acting coagulation factor products, non-factor therapies, and gene therapy—has ushered hemophilia into a new era of treatment. Several of these innovative products have been approved for marketing in China, marking a significant improvement in treatment outcomes for patients with hemophilia. To standardize clinical practices amid these new treatment modalities and evolving therapeutic concepts, the Thrombosis and Hemostasis Group of the Hematology Branch of the Chinese Medical Association and the Chinese Hemophilia Collaboration Group have collaborated to develop relevant guidelines. These guidelines aim to provide guidance for domestic medical professionals engaged in hemophilia diagnosis and treatment, ensuring the delivery of standardized, high-quality care.

血友病是一种Ⅹ染色体连锁的隐性遗传出血性疾病，由凝血因子Ⅷ/Ⅸ（FⅧ/Ⅸ）基因（F8/F9）发生突变导致患者体内FⅧ/Ⅸ缺乏所致，属于国家第一批罕见病目录病种。患者临床表现为自发性或者外伤后出血。主要出血部位为关节和肌肉。出血关节主要为膝关节，其次为踝、髋、肘、腕及肩关节。肌肉出血多见于负重的肌肉群（髂腰肌、腿部、臀部等）。颅内出血不常见，但是死亡率高。

自《血友病治疗中国指南（2020年版）》[1]发表以来，长效凝血因子产品、非因子类产品和基因治疗产品相继问世，部分产品已经在我国上市。这些产品进一步改善了血友病患者的疗效。但同时，这些新型药物对于血友病诊疗的传统评价方法，尤其是临床常规凝血因子活性检测提出了挑战。本版指南结合上述进展，重点对治疗方式分类及定义、新型血友病治疗药物的使用和监测提出具体建议，旨在为医务人员的血友病诊疗实践提供指导，为药品监管部门的药物评价以及医保部门的政策制订提供支持。

本指南所诊疗的对象除所有男性血友病患者以外，也包括轻型、中间型、重型女性血友病患者（FⅧ/Ⅸ活性分别为>5％～40％、1％～5％、<1％）以及有出血表型和无出血表型的携带者（FⅧ/Ⅸ活性≥40％）[2]。

一、指南制订过程

2024年4月，中华医学会血液学分会血栓与止血学组以及中国血友病协作组成员组成专家组。指南制定之初，检索PubMed、万方数据库、维普、中国知网等中英相关文献，检索时间为建库至2025年5月，根据发表时间、发表期刊的质量及内容相关度，最终纳入49篇文献。专家组成员经过多轮讨论形成指南初稿，进一步扩大讨论范围后，基于Delphi法形成推荐意见，采用问卷调查的方式给出推荐强度。推荐专家占比>85％定义为强推荐，75％～84％为推荐，65％～74％为弱推荐。

二、治疗原则

1. 治疗的基本目标是保护关节，避免残疾以及避免严重危及生命的出血事件如颅内出血等的发生，最终的理想目标是让血友病患者与健康人享有一样的生活质量。

2. 建立血友病分级诊疗体系，让患者得到及时且规范的诊疗。

3. 建立血友病多学科合作团队，为患者提供全方位综合关怀及长期随访管理。

4. 应接受经过血友病专业培训的医护人员的治疗。

5. 提升实验室诊断（包括基因检测）的能力，为治疗方案的选择和疗效判断提供检测依据。

6. 为患者提供安全有效的替代治疗以及前沿的治疗方法与理念。

7. 制订并推广能监测血友病患者病程并衡量疾病及其治疗结局（即止血治疗的有效性和相关并发症）的特定工具，如关节超声、药代动力学计算工具等。

8. 急性出血时应尽快到附近的专业医疗机构接受替代治疗等止血治疗或者在家庭进行凝血因子自我注射[3]。出血时应及时给予足量的临时替代治疗。进行手术或者其他创伤性操作时，也应进行充分的临时替代治疗[1]。

9. 提倡在条件允许时进行家庭治疗，但必须由专业人员密切监管，且只有在患者及其家属得到充分培训后才能进行[1]。在家庭治疗期间，患者应定期到血友病中心进行病情评估及治疗咨询。

10. 积极管理血友病治疗相关并发症包括抑制物、骨骼肌肉并发症以及输血相关的病毒感染等。

11. 积极治疗并存病，尤其是糖尿病、高血压等疾病。

12. 患者应尽量避免肌肉注射，必要时（如疫苗接种）需做好临时替代治疗。原则上避免阿司匹林或非甾体类解热镇痛药（某些COX-2抑制剂除外）以及所有可能影响血小板功能的药物。对乙酰氨基酚可用于血友病患者的镇痛；必须应用时（如共患川崎病必须应用阿司匹林）需做好临时替代治疗。

13. 女性血友病患者处理原则与男性患者相同。凝血因子活性高于40％的女性携带者发生异常出血时，应接受血友病中心的专业医疗指导。

三、治疗的分类

血友病的治疗包括针对凝血障碍的治疗和并发症/并存病的治疗。随着非因子类药物广泛使用及基因治疗的不断发展，此指南对针对凝血障碍的治疗进行了重新定义和分类，分为替代治疗（包括凝血因子替代治疗和非因子治疗）以及基因治疗，替代治疗又根据治疗方式分为临时替代治疗［（又称按需治疗（on-demand treatment, episodic treatment）］以及规律替代治疗［又称预防治疗（prophylactic treatment, prophylaxis）］。

（一）替代治疗

1. 临时替代治疗

临时替代治疗是指有出血、外伤后有出血风险、围手术期和其他有创操作时以及康复治疗等过程中给予的临时/短期替代治疗。目的是为了及时止血以及避免手术、康复治疗和其他有创操作中可能的出血。

出血时治疗原则是尽早、足量、足疗程给药直至出血症状停止。及时充分的治疗不仅可以及时止血止痛，更可阻止危及生命严重出血的发生。治疗时凝血因子具体剂量以及持续时间可参见《血友病治疗中国指南（2020年版）》[1]。此种治疗方式无法阻止反复出血导致关节残疾的发生，适合较少发生出血事件的轻型和中间型血友病患者，以及重型血友病患者在规律替代治疗中发生的突破性出血。发生关节以及肌肉出血后，在临时替代治疗基础上，可采取辅助治疗措施（如局部保护、休息制动、冰敷、压迫），肢体出血还可以抬高患肢，以缓解症状和减少出血。关节出血时，非必要，不建议常规关节穿刺抽血减压[4]。

围术期即手术前、手术中和手术后伤口愈合期进行的替代治疗，目的在于手术过程中止血以及促进术后的伤口愈合。凝血因子替代治疗具体方案可参见《血友病治疗中国指南（2020年版）》[1]及《中国血友病骨科围手术期管理指南》[5]。

康复期的替代治疗主要指在康复期间为了避免患者的出血所采取的临时替代治疗方式。详细参见《药物代谢动力学指导下的血友病康复个体化管理—基于改良Delphi法制订的中国专家共识》[4]以及《中国血友病管理指南（2024版）》[6]。

在凝血因子药物选择上，血友病A首选基因重组FⅧ制剂或者病毒灭活的血源性FⅧ制剂，仅在需要紧急止血而又无法获得上述凝血因子药物时可暂时选用冷沉淀或新鲜冰冻血浆等。理论上，每输注1 IU/kg体重的FⅧ可使体内FⅧ活性提高2％。标准半衰期（standard half-life, SHL）FⅧ在体内的半衰期为8～12 h，要使体内FⅧ保持在一定水平需每8～12 h输注1次。血友病B首选基因重组FⅨ制剂以及病毒灭活的血浆源性FⅨ制剂，仅在需要紧急止血、无上述条件时可暂时选用选择凝血酶原复合物（PCC）或新鲜冰冻血浆。每输注1 IU/kg体重的FⅨ理论上可使体内FⅨ活性提高1％，SHL-FⅨ在体内的半衰期约为18～24 h，要使体内FⅨ活性保持在一定水平需每日至少输注1次。

延长半衰期（extended half-life, EHL）的凝血因子与SHL凝血因子相比，可改善半衰期/清除率（FⅧ半衰期延长1.5～1.8倍，FⅨ半衰期延长3倍以上）。EHL产品的研发使用了不同的技术，包括与聚乙二醇的结合以及与其他蛋白质（如白蛋白、IgG1的Fc片段）的融合。目前新研发的另外一种EHL的FⅧ浓缩物rFⅧFc‐VWF‐XTEN应用多种延长半衰期的技术、使其半衰期比SHL-FⅧ产品长3～4倍。在相似给药方案下，与SHL制剂相比，EHL制剂可延长保护时间，减少手术、康复理疗等过程中的出血风险。

2. 规律替代治疗

规律替代治疗是指规律性输注凝血因子或者接受非因子制剂，以减少出血发生，维持正常的关节和肌肉功能为目的的治疗。

（1）接受治疗的对象

一般而言，患者基础凝血因子水平与出血的表型密切相关，凝血因子水平越低的血友病患者，出血表型往往越重。通常情况下，重型患者出血表型最重。部分中间型患者尤其是凝血因子活性1％～2％的患者，出血表型可与重型患者类似[7]–[8]。研究发现，如不接受规律替代治疗，几乎所有重型患者以及部分凝血因子活性1％～2％的中间型患者都会存在至少一个靶关节以及不同程度的关节活动受限。因此对于所有重型患者以及反复发生出血的中间型患者应尽早进行规律替代治疗。对于再次出血风险高的危及生命出血如颅内出血患者，应在出血控制后即开始规律替代治疗。对于反复出血、已经合并关节损伤的儿童或者成人患者，无论血友病严重程度如何，均应该接受规律替代治疗。

（2）治疗开始的时机

根据治疗开始的时机通常分为：①初级规律替代治疗：治疗开始于第2次关节出血前，一般年龄小于3岁，尚未存在关节病变的明确证据（查体或影像学检查）。②次级规律替代治疗：已有≥2次关节出血，但查体或影像学检查尚未发现关节病变时开始的治疗。次级规律替代治疗开始时，患者可能已经存在亚临床关节损伤。③三级规律替代治疗：查体或影像学检查证实存在关节病变后才开始规律替代治疗。

初级和次级规律替代治疗远期疗效更好，充分的初级规律替代治疗可使患者始终维持正常的骨骼以及关节状态，次级规律替代治疗可使患者发生骨骼肌肉病变的风险最小化。初级和次级规律替代治疗均可减少儿童脑出血的发生率[9]。接受三级规律替代治疗的患者，患者关节出血频率以及关节慢性疼痛改善明显[10]，尽管关节磁共振（MRI）检查可能无好转，但关节损害进展减缓、靶关节改善[11]，生活质量提高，也可恢复正常的社会功能。同时，随着非因子替代治疗产品的广泛应用，规避了重型婴幼儿患者应用凝血因子替代治疗的频繁静脉穿刺、静脉通路建立以及抑制物的三大困境，使得开展初级规律替代治疗时间前移。

（3）规律替代治疗持续时间

在一项单中心队列研究中，到成年后停止规律替代治疗的患者，10年后关节情况明显差于持续规律替代治疗的患者[12]。因此建议在条件允许时应长期维持规律替代治疗。

（4）规律替代治疗的方案

规律替代治疗分为凝血因子规律替代治疗以及非因子类药物治疗。

1）凝血因子规律替代治疗

①凝血因子剂量固定的规律替代治疗：关于治疗的方案，国际上没有统一的标准。最低剂量为英国1976年在血友病A中使用12 IU/kg每周1次[13]。目前基于强度的SHL制剂的规律替代治疗包括以下三种方案[9]：①大剂量方案：FⅧ 25～40 IU/kg每2日1次，FⅨ 40～60 IU/kg每周2次。②中剂量方案；FⅧ 15～25 IU/kg每周3次，FⅨ 20～40 IU/kg每周2次。③小剂量预防治疗：FⅧ 10～15 IU/kg每2～3日1次；FⅨ 10～15 IU/kg每周2次。鉴于SHL凝血因子半衰期较短，建议规律替代治疗时应尽量在清晨输注凝血因子。

中剂量方案临床结局（包括关节出血次数和关节评分）稍差于大剂量组，但社会参与度和生活质量两组结果相似[14]，而且中剂量方案明显减少治疗费用，因此从长远来看，其效价比不差于大剂量方案。

临床实践表明与临时替代治疗相比，低剂量方案虽然可以明显减少血友病患儿出血，但并不能减少关节病变的发生。

EHL凝血的治疗间隔根据半衰期来调整，例如rFⅧFc‐VWF‐XTEN可每周给药1次[15]。与SHL凝血因子相比，EHL凝血因子定期规律治疗可减少输注频率，降低静脉穿刺的负担，也减少频繁清晨穿刺的压力；可以维持更高因子谷水平，降低亚临床出血的发生。

在治疗过程中，应该严密监测患者的出血以及关节情况，根据具体情况调整规律替代治疗的频率以及剂量，以达到最终避免出血以及保护关节的目的。

②凝血因子个体化规律替代治疗方案：由于患者之间的出血表型、关节状况、凝血因子的药代动力学特点、患者静脉通路、凝血因子制剂供应情况以及个人偏好等因素存在差异，固定剂量的治疗方案并不能满足所有患者的治疗需求。在一定程度上也可能减低患者的治疗依从性。即使是大剂量方案，也不能完全消除患者的关节出血，而如果同一个关节出血超过2～3次可能会产生不可逆的渐进性结构损伤。因此需要制定更有针对性的个体化方案。药代动力学评估是调整规律替代治疗方案以满足血友病A患者治疗目标范围的重要工具。谷水平、半衰期、增量回收率、药物曲线下面积等是药代动力学指导下个体化治疗的重要参数。药代动力学指导的规律替代治疗对于接受低剂量或中等剂量治疗的患者可能有益[16]。为了减少出血，规律替代治疗期间凝血因子谷水平至少要维持1％，但研究发现凝血因子活性谷水平1％～3％不能避免所有的出血，患者仍有发生关节病变的可能。PedNet队列研究结果显示，内源性因子活性超过5％可作为显著降低关节出血率的阈值，而FⅧ活性>15％、FⅨ活性>10％可以在儿科队列中实现零出血的治疗目标[17]。WFH指南推荐维持凝血因子活性谷水平3％～5％或更高以获得更好的长期疗效[9],[18]。

以往在凝血因子供给不足的情况下，曾建议重型血友病A患者避免剧烈运动。但体力活动对血友病A患者改善生活质量、改善肌张力和增加骨强度（并可防止潜在出血）的益处逐渐被认识[9],[19]。此外，体力活动能力对血友病患者的一般健康状况和心理健康的重要性已被证实[20]。随着医疗条件改善，国内患者对运动也逐渐重视。但是，体力活动需以一定凝血因子水平为基础[21]。Iorio等[22]在2017年发表的Delphi共识声明中指出FⅧ活性谷水平3％～5％适合轻度体力活动，5％～15％适用于出血风险较高的体力活动。因此个体化规律替代治疗方案的制定也应该考虑患者的活动情况[4]。

2）非因子规律替代治疗

非因子药物包括因子模拟物以及凝血再平衡药物，主要用作避免或者减少患者出血的发生。非因子类药物主要以皮下注射的方式给药，操作方便，避免了患者反复静脉穿刺的痛苦，可提高患者用药的依从性和生活质量。相较于凝血因子，非因子类药物血药浓度长期稳定，可为患者提供稳定持续的疗效，有助于达到更高的减少出血的目标。

①因子模拟物：包括在我国已经上市的艾美赛珠单抗和尚未上市的Mim8。

a. 艾美赛珠单抗：艾美赛珠单抗是一种人源化双特异性单克隆抗体，通过模拟FⅧa的辅因子功能，可同时桥接FⅨa和FⅩ，使FⅩ在缺乏FⅧ的情况下得以激活，重新恢复凝血通路。推荐的给药方案为前4周给予负荷剂量3 mg/kg每周1次皮下注射，以快速达到目标血药浓度；第5周起给予维持剂量1.5 mg/kg每周1次，也可以3 mg/kg每2周1次或6 mg/kg每4周1次，不同用法疗效类似。艾美赛珠单抗在成人或者儿童包括婴幼儿中均取得良好的疗效[23]–[29]。尽管在亚洲地区的研究中，相同的给药方式，艾美赛珠单抗的药物谷浓度较前期研究低22％左右，但仍在其观察到的范围内[27]。一些临床医师也在探索小剂量的方式。基于真实世界的研究显示，我国大部分患者采用减低剂量艾美赛珠单抗治疗，且取得一定的疗效[30]。但疗效还需要更多的研究以及临床实践去证实。该药已经在国内以及国外多个国家获批用于全年龄段血友病A伴或者不伴FⅧ抑制物患者的规律替代治疗。

在接受艾美赛珠单抗治疗的非抑制物血友病A患者发生突破性出血时，应接受FⅧ替代治疗，由于FⅧ、FX和FⅨa的亲和力高于艾美赛珠单抗[31]，FⅧ可将艾美塞珠单抗从结合位点置换下来，有研究显示当两者同时应用时，暂未发现凝血酶生成的潜能存在叠加的效应。目前已经有FⅧ替代治疗安全用于发生突破性出血的先例，但连续使用FⅧ时需要考虑艾美赛珠单抗的基础止血作用。大剂量FⅧ使用是否会增加血栓的风险仍需要更多的临床数据。

艾美赛珠单抗模拟FⅧa的桥接功能，直接激活凝血级联反应，会影响基于APTT的凝血试验，3～5 µg/ml（理论治疗剂量55 µg/ml）即可使APTT检测正常化。因此接受该药替代治疗的患者，不可依据基于APTT的一期法FⅧ活性去评估艾美赛珠单抗凝血活性或内源性FⅧ活性水平。

标准剂量艾美赛珠单抗治疗的患者可不用定期监测其凝血情况；对减量替代治疗和不规范治疗的患者建议进行药物浓度、等效FⅧ活性水平的检测，或凝血酶生成潜能等以评估其凝血能力，有条件者可进行药代动力学指导个体化减量治疗。艾美赛珠单抗可影响基于人源性发色底物检测试剂，但对于牛源性发色底物检测无影响。艾美赛珠单抗的血浆药物浓度可采用调整样本稀释倍数的一期凝固法进行检测，并以艾美赛珠定标品定标的特异性标准曲线进行分析。判断艾美赛珠单抗的疗效相当于多少FⅧ活性（即等效FⅧ活性）时，建议使用基于人源性发色底物法检测。对FⅧ抑制物阳性血友病患者，推荐使用牛源性发色底物法检测FⅧ抑制物滴度。接受艾美赛珠单抗治疗的血友病A患者在同时使用FⅧ替代治疗时，牛源发色底物法可准确监测SHL/EHL制剂FⅧ活性水平。

b. Mim8（denecimig）：Mim8是一种全人源双特异性IgG4抗体，通过桥接FⅨa和FⅩ模拟FⅧa的功能，增强FⅨa的蛋白水解活性并能够有效激活FⅩ[32]。Mim8和艾美赛珠单抗的作用方式相似，但他们各自的抗FⅨa和抗FⅩ臂存在差异，导致其FⅧa样功能存在差异。FRONTIER1研究显示Mim8耐受性良好，药代动力学以及药效学特性支持从每周到每月的给药方式，初步疗效结果显示，高剂量组大部分（70％～100％）血友病A患者未发生需要治疗的出血事件[33]。Mim8作为第二种FⅧa模拟剂可能让血友病A伴或不伴抑制物患者在减少出血方面获益。Mim8的等效FⅧ活性、输注FⅧ活性及FⅧ抑制物的检测方法同艾美赛珠单抗。

②凝血再平衡药物：另一类非因子类药物作用机制是通过抑制抗凝系统的成分包括抗凝血酶（antithrombin, AT）以及组织因子途径抑制物（tissue factor pathway inhibitor, TFPI）的功能，以恢复凝血平衡，从而达到止血的目的。

a. 抗AT制剂（Fitusiran）：AT是一种重要的血浆蛋白，属于血液凝固系统中的天然抗凝因子，主要由肝脏合成。它的主要功能是抑制凝血酶和其他凝血因子（如FⅩa），起到抗凝的作用。Fitusiran利用RNA干扰机制来切割和降解抗凝血酶mRNA，以减少肝脏抗凝血酶的合成[34]。在≥12岁伴或不伴抑制物的血友病A以及血友病B患者Ⅲ期临床研究中显示出良好的减少出血效果[35]–[36]。在伴或不伴抑制物的血友病A或B患者的Ⅲ期开放标签扩展研究（ATLAS-OLE）中，目标AT活性为15％～35％，年化出血率（ABR）明显低于凝血因子按需治疗、旁路制剂临时治疗以及与旁路制剂定期规律治疗组，与中高剂量凝血因子规律替代治疗组相当[37]。2025年3月28日美国FDA批准Fitusirin用于血友病A或B型（无论是否存在FⅧ或Ⅸ抑制物）的成人及≥12岁青少年患者的规律替代治疗。推荐初始剂量为50 mg每2月1次皮下注射，根据AT水平调整剂量或者给药间隔，维持AT活性15％～35％。

b. 抗TFPI制剂：TFPI是一种糖蛋白和多价Kunitz型丝氨酸蛋白酶抑制剂。包含三个Kunitz型结构域。第一个Kunitz结构域（K1）可抑制组织因子FⅦ（TF-FⅦa）复合物，第二个Kunitz结构域（K2）可抑制FⅩa限制凝血级联反应开始时的凝血激活。Concizumab是一种靶向TFPI的IgG4单克隆抗体，通过抑制TFPI的K2结构域发挥功能。在血友病A和血友病B伴抑制物患者的Ⅲ期临床研究显示，Concizumab治疗组中位ABR优于按需组[38]。在2023年，Concizumab获得加拿大、欧盟、瑞士和美国批准用于≥12岁伴抑制物的青少年和成年血友病A和血友病B患者规律替代治疗，同年获得澳大利亚和日本批准用于伴或不伴抑制物青少年和成年血友病A和血友病B患者的规律替代治疗。Marstacimab（马塔西单抗）是一种全人源单克隆抗体（IgG1），也是通过抑制TFPI的K2结构域发挥功能，马塔西单抗的Ⅱ期临床研究中，所有治疗队列患者平均年化出血率较治疗前明显好转[39]。该药已经在美国和欧盟获批上市，用于≥12岁不伴抑制物的青少年和成年血友病A和B患者的规律替代治疗。马塔西单抗的给药方式为皮下注射，用量为首剂300 mg（分2个部位，每个部位150 mg）的负荷剂量后，开始每周150 mg给药。目前还没有明确的检测方法来测量anti-TFPI抗体水平，可选择检测TFPI的抗原水平和依赖TF的发色底物法检测TFPI活性。

虽然在前期临床试验过程中取得良好的疗效，非因子类药物也存在实验室客观疗效监测的困难等问题。非因子药物治疗前后可进行凝血酶生成试验（thrombin generation assay, TGA）、血栓弹力图及血块波形分析（clot waveform analysis, CWA），通过观察患者止血潜能变化来分析其疗效。但需要注意这些检测并不能提供有关药物的真正的FⅧ等效浓度。且目前多数试剂以及检测方法在国内没有相应的注册证，不能大规模开展检测。非因子类药物在使用过程中需要密切观察血栓相关的症状，尤其是当患者合并先天性/获得性易栓症或者其相关危险因素（糖尿病、高血压等）。

（二）基因治疗

目前血友病的基因治疗方案主要利用腺相关病毒（adeno-associated virus, AAV）载体来递送所需的基因。长期疗效已得到初步验证[40]。美国和欧盟等批准了3款血友病基因治疗药物上市，包括Roctavian[41]、Hemgenix[42]、Beqvez[43]。2022年5月，中国公布了首个AAV靶向肝脏的血友病B基因治疗研究结果，证实了AAV载体在中国人群中的安全性和有效性[44]（该产品已经于2025年4月10日在国内正式上市）。

尽管基因治疗尚存在一些待解决的问题（AAV载体基因片段对宿主基因组的插入诱变风险、宿主针对载体衣壳的免疫反应、长期使用免疫抑制剂带来的相关不良反应、患者体内针对AAV的中和抗体导致基因治疗的人群受限、目的基因是否能长期表达等），但随着科技的进步，基因治疗将会给血友病患者带来治愈疾病的希望。

（三）其他药物治疗

1. 1-去氨基-8-D-精氨酸加压素（DDAVP）

主要用于轻型血友病A，少数中间型血友病A可能也有效。每次剂量为0.3 µg/kg，用50～100 ml生理盐水稀释后静脉滴注，至少30 min滴完，通常在60 min时FⅧ和VWF水平达到峰值。成人可每日1次或每12 h 1次，儿童每日1次，1～3 d为一个疗程，该药多次使用后疗效可能变差，效果不佳时应及时补充FⅧ制剂。需要进行药物预实验以确保治疗效果后再进行临床治疗出血时使用，使用过程中注意限水。该药在幼儿、老年人以及高血压患者慎用，2岁以下儿童以及孕妇禁用。

2. 抗纤溶药物

常用药物有氨甲环酸、6-氨基己酸、止血芳酸等，可作为辅助止血药物使用，但在泌尿系统出血时禁用，避免与PCC同时使用。

推荐：

①在减低出血频率方面，规律替代治疗优于临时替代治疗（强推荐：100％）。

②条件允许时，建议所有重型血友病患者以及凝血因子活性1％～2％的中间型伴反复出血的血友病患者接受规律替代治疗，药物可选择SHL凝血因子、长半衰期凝血因子或非因子药物（强推荐：100％）。

③条件允许时，重型血友病患者应尽早开展规律替代治疗，以初级规律替代治疗最优；已经合并关节损伤者，应尽早开始三级规律替代治疗（强推荐：100％）。

④条件允许时，规律替代治疗的患者可进行个体化治疗，应综合考虑患者的出血表型、关节状态、个体PK特点、体力活动、经济条件以及个人偏好等各种因素（强推荐：100％）。

⑤使用非因子类药物进行规律替代治疗时，血友病A患者可选择FⅧ模拟物或者凝血再平衡药物；血友病B患者可选择凝血再平衡药物（强推荐：100％）。

⑥非因子类药物需在有经验的医护人员指导下使用；开始治疗前，须对患者进行教育（包括药物作用方式/对常规凝血试验的影响、给药途径和储存、突破性出血或手术时的治疗策略等）。需严密监测血栓形成的风险，特别是遗传性或获得性易栓症或伴有糖尿病、高血压等相关危险因素（强推荐：100％）。

⑦评估艾美赛珠单抗等效FⅧ活性水平时，推荐用人源性发色底物法试剂来进行检测；进行FⅧ抑制物监测时，推荐使用牛源性发色底物法试剂检测（强推荐：96.2％）。

⑧基因治疗需在有相关经验的血友病中心开展。患者在接受基因治疗后应密切监测凝血因子水平以及转氨酶变化至少1年，并及时调整免疫抑制剂方案（强推荐：100％）。

四、物理治疗和康复

物理治疗与康复可以预防、减轻和减少肌肉关节的功能障碍，提升日常生活活动能力和生活质量，是综合治疗的重要组成部分。详细参见《药物代谢动力学指导下的血友病康复个体化管理—基于改良Delphi法制定的中国专家共识》[4]以及《中国血友病管理指南（2024版）》[6]。

推荐：

①急性出血的患者，建议在专业人员指导下确定出血停止后尽早开始物理治疗（强推荐：100％）。

②物理治疗和康复训练等均应由经过培训的专业人员负责实施或在其指导下实施（强推荐：100％）。

③功能评估应由经过培训的专业人员负责实施（强推荐：100％）。

五、并发症的处理

（一）抑制物的处理

血友病患者发生抑制物时，治疗原则包括控制出血和清除抑制物。

伴抑制物血友病患者发生出血后，根据抑制物滴度以及出血的严重程度，可采取旁路途径药物［如基因重组活化FⅦ（rFⅦa）、PCC］或加大凝血因子输注剂量的方式止血。非因子药物定期规律替代治疗可有效减少伴抑制物患者的出血。

免疫耐受诱导治疗（immune tolerance induction, ITI）是清除血友病抑制物最有效的治疗。可根据患者抑制物滴度，采取不同ITI治疗剂量：抑制物滴度小于200 BU/ml的患者，可采取小剂量ITI治疗，比如25～50 IU/kg每日1次或隔日1次；疗效不佳时可考虑联用免疫抑制剂或加大ITI治疗剂量。若抑制物滴度≥200 BU/ml，则需要采取中剂量（100 IU·kg^−1^·d^−1^）或大剂量ITI治疗（200 IU·kg^−1^·d^−1^），必要时可联用免疫抑制治疗。参见《血友病合并抑制物诊断与治疗中国指南（2023年版）》[45]及《中国血友病管理指南（2024版）》[6]。

（二）血友病性关节病的处理

血友病性关节病是指由于反复关节出血导致关节功能受损或关节畸形。血友病性关节病的发展过程往往始于关节出血，出血导致慢性滑膜炎，进而发展到软骨受损，逐渐进展可造成骨质破坏导致关节畸形。关节病治疗的目的是减少关节出血、改善关节功能、缓解疼痛以及帮助患者恢复正常的日常活动。详细治疗方案请参见《中国血友病骨科手术围手术期管理指南》[5]。

（三）血友病性假瘤的处理

血友病性假瘤是发生在血友病患者中的一种少见但严重的并发症。它的本质是发生在肌肉或骨骼的一种囊性包裹的血肿，通常是由于血友病患者发生出血后凝血因子替代治疗不充分，长期慢性出血的结果。

假瘤治疗的目标应是彻底清除假瘤并尽可能重建正常的解剖结构。清除假瘤最理想的方法是完整切除。具体参见《中国血友病骨科手术围手术期管理指南》[5]。

（四）血液传播性感染

目前常见的血液传播性病毒为人类免疫缺陷病毒、丙型肝炎病毒、乙型肝炎病毒等。这些病毒感染后，除了可能导致免疫缺陷和肝硬化外，还可导致肿瘤的发生率增加。早期的血友病患者很多感染了血液传播性病毒。目前国内上市的凝血因子产品均经过病毒去除或灭活，且原料血浆实行检疫期管理，具有较好的安全性。冷沉淀未进行有效的病毒灭活，核酸检测可大大缩短病毒感染的窗口期，但仍不能完全排除病毒感染风险。建议定期进行病毒血清学筛查，一旦罹患有关感染，建议患者在血友病综合关怀团队的指导下进行抗病毒治疗。

（五）口腔健康的维护

良好的口腔卫生是预防牙龈出血和牙周疾病的核心措施。建议每日至少刷牙2次，采用软毛牙刷及“巴氏刷牙法”，避免用力过猛损伤牙龈，并配合含氟牙膏及氯己定漱口水以减少牙菌斑。牙线和冲牙器可用于清洁牙缝，但需谨慎操作以防黏膜损伤。儿童患者应在第一颗乳牙萌出时（约6月龄）接受口腔检查，成人每半年至1年需进行1次专业评估，以便早期发现龋齿、牙周炎等问题。口腔治疗需在血液科医生指导下进行，必要时提前输注凝血因子或使用抗纤溶药物（如氨甲环酸）以降低出血风险。若需拔牙或手术，应确保凝血因子活性水平达标（如凝血因子活性>30％），术后采用局部压迫、冷敷及纤维素基质保护创面，并密切监测延迟性出血的发生。此外，饮食上需避免过硬或刺激性食物，以减少口腔黏膜损伤[9]。

（六）血栓相关疾病

虽然血友病以自发性或创伤后的出血为主要临床表现，但无论动脉血栓性疾病还是静脉血栓栓塞症均可发生于血友病患者。血友病患者血栓处理详见《中国血友病管理指南（2024版）》[6]。

六、血友病关节功能评估及生活质量评估

定期对血友病患者进行关节功能评估可以为制定或调整预防治疗方案以及处理关节病变提供依据。另外，在定期规律替代治疗过程中，有些患者可能发生不能感知的亚临床出血，这些出血也可导致慢性关节损伤。对这些损伤的识别也有助于调整规律替代治疗方案达到最终保护关节的目的[46]。

血友病性关节病的影像学评估是监测血友病替代治疗疗效、关节病进展和防止严重关节并发症的主要手段。检查方法包括X线、MRI和超声。其中，超声检查经济、简便和实时，能够探测血友病性关节病的关节积液、滑膜增生和关节浅表部位软骨破坏，Doppler超声能够显示急性期滑膜血流信号增加，适用于筛查和监测疾病进展。目前推出多种超声检查的评分系统，2015年世界血友病联盟推荐首选HEAD-US。2018年中国血友病协作组[47]发表针对我国血友病患者进行优化的超声评估量表HEAD-US-C。HEAD-US-C在HEAD-US的基础上增加了关节积液和滑膜血管的评估，提高了血友病A急性期疾病活动性超声评价的客观性和敏感性，方便对关节进行快速和简单的评估。血友病性关节病超声评估专家共识（2022年版）将HEAD-US-C作为中国首选的超声评估系统，用于筛查和随访血友病关节病变[47]。MRI是目前公认的诊断血友病性关节病的最敏感方法，具有多参数、多序列、多方位成像和软组织分辨率高的特点，不仅能显示关节积液不同时期的出血改变、滑膜增生和含铁血黄素沉积，而且能够早期显示软骨异常[1]。缺点是费用高、设备不普及、检查费时、疾病本身因素如含铁血黄素大量沉积时MRI图像会产生磁敏感伪影、幼儿检查时需要镇静等。

近年来发表的各种评估量表为关节功能和生活质量评估提供了可以量化的工具，建议采用经过验证的量表如HJHS中文版、成人血友病活动列表（hemophilia activities list, HAL）、CHO-KLAT中文版以及SF-6Dv2中文版[48]等。

七、疫苗接种的问题

传染病预防性接种至关重要。迄今无证据表明疫苗接种会诱发抑制物产生。血友病患者应接受其年龄阶段推荐的所有疫苗接种，接种方式首选口服，其次皮下注射，尽量避免肌肉注射。如无法以其他接种方式替代肌肉注射，可提前输注凝血因子预防出血。接受足量艾美赛珠单抗预防治疗的儿童，肌肉注射疫苗前无需提前输注凝血因子。建议使用最小规格的针头（25～27号）进行免疫接种。疫苗接种前注射部位冰敷5 min，接种后注射部位按压至少10 min，有助于减少出血和肿胀。无论皮下还是肌肉注射，疫苗接种后2 d内均需密切观察注射部位，如有血肿产生，按压及冰敷处理30 min后仍进行性增大，则需输注凝血因子止血[9],[49]。
